# Protocol for preparing natural peptidoglycan nanoparticles and purifying plant-derived antigens

**DOI:** 10.1016/j.xpro.2025.104260

**Published:** 2025-12-07

**Authors:** Hui-Xin Meng, Shi-Yu Ren, Xue-Jiao Xu, Yong-Feng Guo, Hai-Ping Diao, Shi-Jian Song

**Affiliations:** 1 Qingdao Municipal Key Laboratory of Plant Molecular Pharming, Tobacco Research Institute, Chinese Academy of Agricultural Sciences, Qingdao 266101, China; 2 Beijing Life Science Academy (BLSA), Beijing 102200, China

**Keywords:** plant sciences, protein biochemistry, biotechnology and bioengineering

## Abstract

Purifying plant-derived proteins is challenging due to host contaminants and high downstream costs. Here, we present a protocol for the preparation of natural peptidoglycan nanoparticles (NPNs) from lactic acid bacteria (LAB) and their use to selectively capture SCpl7-tagged antigens from plant extracts. We describe steps for NPN preparation, plasmid construction and infiltration, and total soluble extraction. We then detail procedures for purifying target proteins. This protocol enables rapid protein capture, offering a cost-effective alternative to conventional Ni^2+^-NTA purification.

For complete details on the use and execution of this protocol, please refer to Song et al.^1^ and Song et al.^2^

## Highlights

• Steps for preparing natural peptidoglycan nanoparticles (NPNs) from *lactic acid bacteria*
• Procedures for plasmid construction and *N. benthamiana* leaf infiltration
• Guidance on extracting total soluble proteins from infiltrated plant tissues
• Instructions for NPN-mediated purification of recombinant proteins

## Abstract (editor highlights)

Publisher’s note: Undertaking any experimental protocol requires adherence to local institutional guidelines for laboratory safety and ethics.

## Graphical abstract

**Figure:**
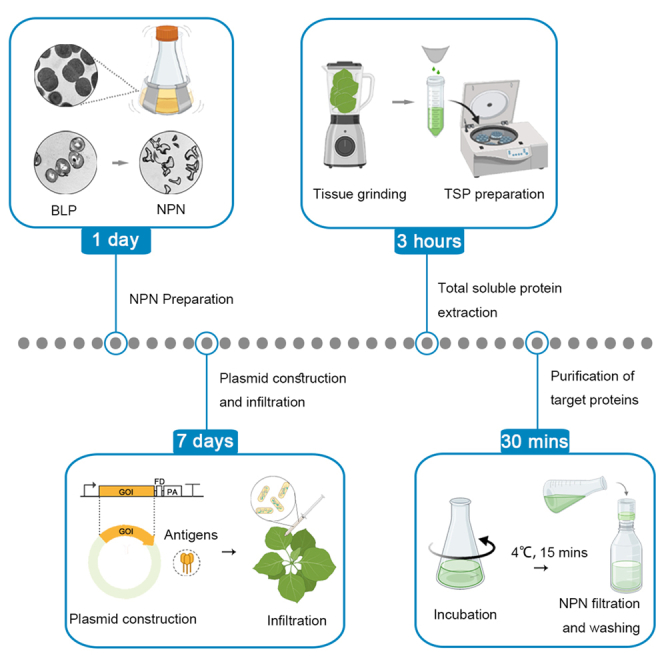

## Before you begin

### Innovation

Plant molecular farming is an attractive platform for producing vaccines, antibodies, and enzymes due to its safety, scalability, and low cost.^3^ Plants are free of human pathogens and endotoxins, grow in simple greenhouse conditions, and can generate millions of vaccine doses in few weeks.^4^ However, the downstream purification of recombinant proteins remains a major bottleneck. For example, extraction of plant-derived proteins releases endogenous proteases that can degrade the target protein over time, while host proteins and secondary metabolites may non-specifically bind to affinity resins or induce aggregation.^5^ Together, these issues substantially reduce yield and purity, increasing the cost and complexity of plant-based protein purification. It has been reported that downstream processing (DSP) represents the most challenging and cost-intensive step in plant-based recombinant protein production, accounting for up to 80% of total production costs.^6^ There is a critical need for plant-adapted, scalable purification technologies that leverage novel materials to enhance efficiency and yield.

To address the challenges, we previously developed bacterium-like particles (BLPs) by inactivating *Lactococcus lactis* through TCA heat treatment, which effectively removed surface proteins and lipids while preserving the peptidoglycan backbone. The ectodomains of avian influenza virus hemagglutinins (HAs) were genetically fused to the cell wall–binding domain LysM from the endolysin AcmA to generate the recombinant protein HA-LysM. Although HA-LysM was efficiently expressed in plants, its binding affinity to BLPs was limited. Previous studies have shown that the binding efficiency of LysM is critically dependent on the integrity of its three tandem repeats, as either deletion or addition of repeats can markedly alter its affinity toward *Lactococcus* cell walls.^7^ Based on this principle, a trimerization domain (mCor1) was introduced between HA and LysM to construct HA-mCor1-LysM, which enabled homologous trimerization of LysM and substantially enhanced its binding capacity to BLPs. In immunization experiments in mice and chicken, these BLPs induced strong immune responses without the need for exogenous adjuvants. Furthermore, when two distinct trimeric HA antigens (H5N6 and H9N2) were either co-displayed on a single BLP or administered as separate BLP formulations, robust and balanced immune responses against both antigens were achieved.^1^

Although BLPs enabled the purification and delivery of plant-derived recombinant protein vaccines, transmission electron microscopy revealed that residual subcellular components and endogenous proteins remained within the particles, raising safety concerns for their *in vivo* use as delivery vehicles. To address this issue, we subjected BLPs to enzymatic digestion with various proteases and found that trypsin treatment effectively removed the residual subcellular structures and proteins, yielding purified natural peptidoglycan nanoparticles (NPNs). Through systematic screening of trimerization and cell wall–binding domains, the combination of the FD trimerization motif and the SCpl7^8^ binding domain was identified as optimal, markedly enhancing the expression of full-length recombinant proteins and improving their binding capacity to NPN from 2 μg/unit to 7 μg/unit. Additionally, NPN-conjugated recombinant antigens exhibited a sustained release profile following intramuscular injection in mice, eliciting a more robust immune response.^2^ This protocol outlines the preparation and application of NPNs for purifying and delivering plant-derived antigens.

### Plant culture

**Timing: 4 weeks**

Here, we describe steps for plant cultivation, including details on growth cycle, temperature, and light conditions.

1. Sow *Nicotiana benthamiana* (*N. benthamiana*) seeds regularly on moist soil.
2. Cover with a plastic wrap to retain moisture for 1 week in greenhouse.

***Note:*** Temperature: 25°C; Photoperiod: 16 h light / 8 h dark; Light intensity: ∼100–150 μmol m^−2^ s^−1^.

3. Transplant seedlings (7–10 days) into 8–10 cm diameter pots with fresh potting soil consisting mainly of peat, with perlite and vermiculite as supplements.

***Note:*** Handle seedlings gently by the cotyledons to avoid damaging the stem. Keep covering with a plastic warp for 4–7 days.

4. Remove the plastic wrap on 14 days post sowing, water every 2–3 days depending on humidity until 4 weeks old of the plants.

***Note:*** Do not water the plants the day before infiltration. We found that *N*. *benthamiana* plants freshly watered were more difficult to infiltrate, with reduced efficiency of infiltration solution entry. This phenomenon is likely due to cell swelling after water uptake, which reduces intercellular space. Therefore, we recommend avoiding watering the plants on the day before infiltration.

### LAB culture

**Timing: 1 day**

Here, we describe steps for LAB cultivation, specifying the strain, growth medium, incubation temperature, and duration.

5. Retrieve LAB strain (*L. sakei*) from glycerol stock or agar slant.
6. Aseptically inoculate 500 μL of thawed glycerol stock into 15 or 50 mL MRS broth (Dissolve 51 gram in 1 L distilled water and add 0.1% Tween 80) in a sterile falcon tube.
7. Incubate the tube under 37°C for 12–16 hours for exponential growth.

***Note:*** For optimal growth of *L. sakei*, which thrives under anaerobic conditions, fill the tube with MRS broth to minimize the air space. Keep the tube stationary during incubation and avoid shaking.

## Key resources table

**Table:** 

REAGENT or RESOURCE	SOURCE	IDENTIFIER
**Antibodies**		

Mouse monoclonal anti-His	Novus	AD1.1.10

**Bacterial and virus strains**		

*Lactic Acid Bacteria*	*L. sakei*	N/A
*Agrobacterium*	GV3101	N/A
*E. coli*	DH5α	N/A

**Biological samples**		

*Nicotiana benthamiana*	N/A	N/A

**Chemicals, peptides, and recombinant proteins**		

LB Broth	LPS Solution	Cat#LBL-05
MRS Broth	Millipore	69966-500G
Kanamycin	Solarbio	K8020
Rifampicin	Solarbio	R8011
Tween 80	Merck	P1754
Restriction Endonuclease	NEB	N/A
rCutsmart	NEB	N/A
Gel extraction kit	TIANGEN	DP219-02
T4 Ligase	Takara	2011A
MgCl_2_	Sigma	208337
MES	VETEC	145224-94-8
Acetosyringone	Solarbio	A8110
Trichloroacetic acid	SCR	76-03-9
Trypsin	Sigma	T2600000
NaCl	SCR	7647-14-5
Tris-HCl (PH=7.5)	Sangon Biotech	B548124-0500
PMSF	Solarbio	P8340
Tween 20	Sigma	P1379
EDTA	Solarbio	E1170

**Critical commercial assays**		

Plasmid Mini-Prep kit	TIANGEN	DP103

## Materials and equipment

**Table:** Plant infiltration buffer

Reagent	Final concentration
MgCl_2_	10 mM
MES	10 mM
Acetosyringone	100 μM

Prepare the solution according to the required amount of *Agrobacterium*, using double-distilled water. Store the prepared solution at room temperature.

**Table:** Protein extraction buffer

Reagent	Final concentration
Tris (pH 7.5)	50 mM
NaCl	150 mM
EDTA	1 mM
Tween 20	0.1%
PMSF	1 mM

Prepare the protein extraction buffer according to the amount of plant tissue, store it on ice or at 4°C, and add PMSF immediately before mixing with the tissue.

## Step-by-step method details

### NPN preparation

**Timing: 3–5 h**

**Figure 1:**
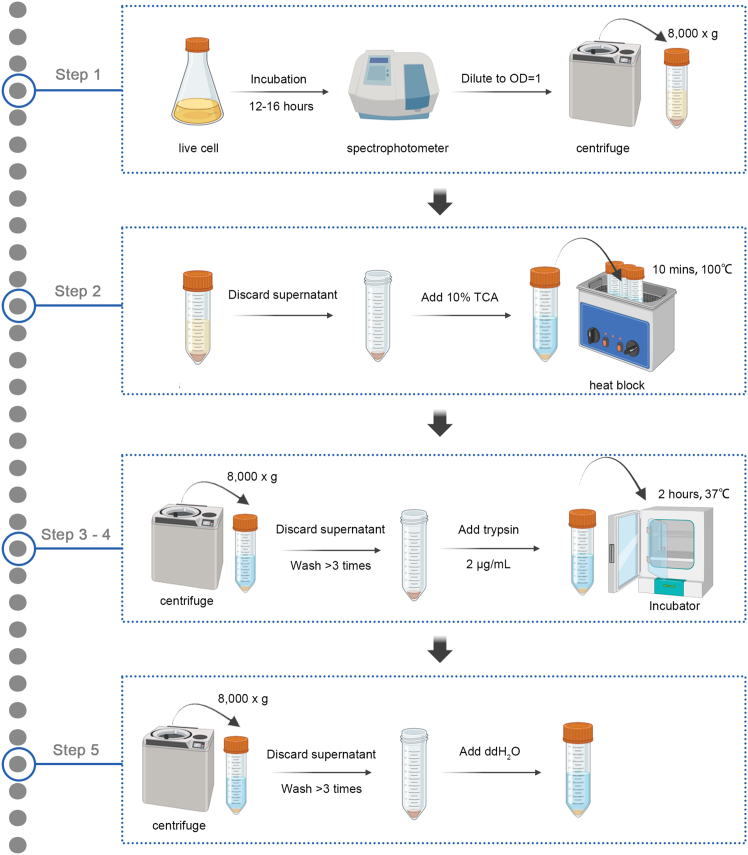
Flow chart of steps involved in NPN preparation

Here, we describe steps for NPN preparation, specifying the TCA treatment, washing, and enzymatic digestion procedures used to convert *L*.*sakei* cells into NPNs (Figure 1).

1. LAB cells collection:
  a. Determine the optical density (OD) of the bacterial culture.
  b. Collect the bacterial cells by centrifugation at 8,000 × *g* for 10 minutes at room temperature.
2. TCA treatment:
  a. Discard the supernatant carefully.
  b. Resuspend the bacterial pellet in 10% (w/v) TCA solution. Use sufficient volume to fully immerse the pellet (e.g., 5 mL TCA for 10 mL cell culture).
  c. Incubate the suspension at 100°C for 10 minutes using a water bath or heating block.
  d. Cool the suspension to room temperature.

***Note:*** TCA treatment removes extracellular macromolecules from LAB cells, including proteins and lipids. At this stage, these inactivated LAB cells are referred to as bacterium-like particles (BLPs).^9^

3. Washing out of remaining TCA:
  a. Centrifuge at 8,000 × *g* for 10 minutes to pellet the BLPs.
  b. Discard supernatant carefully.
  c. Resuspend the BLPs in an appropriate volume of TBS buffer.

***Note:*** Repeat the washing step 3 times: centrifuge → discard supernatant → resuspend in TBS. Purpose: Remove residual TCA completely.

4. Trypsin treatment:
  a. Resuspend the washed BLP pellet in Tris buffer (50 mM, pH 7.8) supplemented with trypsin at 2 μg/mL.
  b. Incubate the suspension at 37°C for 2 hours to allow enzymatic digestion.

***Note:*** SDS-PAGE and BFA analyses revealed that trypsin treatment effectively degraded residual intracellular proteins within the BLPs. Cryo-TEM imaging further confirmed the removal of remaining subcellular structures, yielding peptidoglycan shells with diameters ranging from approximately 100 to 300 nm. At this stage, these particles were designated as natural peptidoglycan nanoparticles (NPNs).^2^

5. Washing out of remaining Trypsin:
  a. Centrifuge at 8,000 × *g* for 10 minutes to pellet the NPNs.
  b. Discard the supernatant carefully.
  c. Resuspend the NPNs in an appropriate volume of TBS buffer.
  d. Resuspend the final NPN pellet in an appropriate buffer or sterile distilled water at a concentration of 1 unit for downstream applications (where 1 unit corresponds to the amount of NPN derived from 1 mL of *L. sakei* culture at OD_600_ = 1).

***Note:*** Wash the pellet 3 times with sterile ddH_2_O to remove residual trypsin: resuspend → centrifuge → discard supernatant.

**CRITICAL:** Advice on sample handling and storage: short term storage (If the NPNs are to be used within few weeks, please store them at 4°C); longer term storage (If the NPNs are to be used several months later, sterilize them in an autoclave and store sealed at room temperature).

### Plasmid construction and infiltration

**Timing: 7 days**

Here, we describe steps for recombinant protein expression in *N*. *benthamiana*, specifying the gene cloning, *Agrobacterium* transformation, plant infiltration and post-infiltration incubation procedures.

6. Plasmid construction:
  a. Digest synthesized gene fragment (GFP/HA/Spike-FD-SCpl7) and vector (pTEX1L) with compatible restriction enzymes (*BamH*I and *Xho*I).
  b. Purify digested fragments via gel extraction kit.
  c. Ligate the GFP/HA/Spike-FD-SCpl7 into the vector using T4 DNA ligase.
  d. Transform ligation product into competent *E. coli* DH5α cells by heat shock.
  e. Plate on LB agar with appropriate antibiotics; incubate overnight at 37°C.
  f. Screen colonies by colony PCR or restriction digest for positive clones.
  g. Confirm correct sequence by Sanger sequencing.
  h. Prepare high-quality plasmid DNA (Mini-Prep) for *Agrobacterium* transformation.
7. *Agrobacterium* transformation.
  a. Thaw competent *Agrobacterium* cells on ice.
  b. Mix 100 ng plasmid DNA with 50 μL competent cells and keep on ice for 2 minutes.
  c. Heating the DNA and competent *Agrobacterium* cells mixture for 1.5 minutes and then keep it on ice for 5 minutes.
  d. Add 1 mL LB medium and incubate at 28°C, shaking, for 2 hours.
  e. Plate 100 μL culture on LB agar containing appropriate antibiotics.
  f. Incubate plates at 28°C for 2 days.
  g. Pick single colonies for confirmation by colony PCR.
8. Plant infiltration.
  a. Inoculate a single colony of transformed *Agrobacterium* into 5 mL LB medium with antibiotics.
  b. Grow overnight at 28°C, 200 rpm.
  c. Transfer 1 mL overnight culture into 50 mL LB with antibiotics.
  d. Grow until OD_600_ = 0.8–1.0.
  e. Harvest cells by centrifugation at 4000 × *g* for 10 minutes at room temperature.
  f. Resuspend pellet in plant infiltration buffer to a final OD_600_ = 0.5–1.0.
  g. Incubate resuspended *Agrobacterium* at room temperature for 1–2 hours to induce virulence genes.
  h. Using a needleless syringe, infiltrate the *Agrobacterium* suspension into the abaxial surface of fully expanded *N. benthamiana* leaves by gently pressing the syringe against the leaf and applying pressure until the leaf area becomes water soaked.

***Note:*** If a larger amount of protein is required, more *N. benthamiana* plants need to be infiltrated. In this case, a vacuum infiltration system should be employed.

9. Post-infiltration incubation.
  a. After infiltration, grow plants under normal growth conditions (22°C–25°C, 16 hours light/8 h dark).
  b. Monitor recombinant protein expression typically after 3 days post infiltration using western blot and harvest it for further experiments.

### Total soluble protein extraction

**Timing: 1–3 h**

Here, we describe steps for total soluble protein extraction from infiltrated *N*. *benthamiana* leaves, specifying the tissue harvesting, homogenization, and clarification procedures used to obtain protein extracts for subsequent NPN purification.

**CRITICAL:** Prior to NPN purification, recombinant protein expression levels should generally be assessed using western blotting and Coomassie Brilliant Blue staining. Although no correlation between NPN binding capacity and protein expression level was observed, achieving expression levels of approximately 100 mg/kg or higher is typically necessary to maintain cost-effective plant-based production.

10. Tissue homogenization.
  a. Harvest the infiltrated *N. benthamiana* leaf tissue.
  b. Immediately freeze samples in liquid nitrogen.
  c. Grind frozen leaf tissue to fine powder using mortar and pestle pre-chilled with liquid nitrogen.

***Note:*** Store the tissue powder at −80°C if not processing immediately.

11. Soluble Protein Extraction.
  a. Transfer powdered tissue to a pre-chilled tube.
  b. Adding protein extraction buffer at a ratio of about 10 mL per gram of leaf tissue.
  c. Homogenize thoroughly by using a homogenizer to ensure complete protein solubilization.
  d. Centrifuge the homogenate at 12,000–15,000 × g for 10 minutes at 4°C to pellet insoluble debris.
  e. Carefully collect the clear supernatant containing total soluble proteins (TSP) into a fresh chilled tube on ice for further experiments.

***Note:*** In large preparations, to ensure complete removal of fine debris, the supernatant should be subjected to centrifugation at least three times consecutively. Prior to each subsequent centrifugation, the collected supernatant must be filtered through Miracloth to minimize carryover of precipitates, thereby optimizing the efficiency of the following centrifugation step.

### Purification of target proteins

**Timing: 30 min**

Here, we describe steps for NPN-mediated protein purification, specifying the filtration, incubation, and washing procedures required to obtain sterile and high-purity recombinant proteins suitable for *in vivo* or *in vitro* applications.

12. Pass the final supernatant through a sterile 0.22 μm bacterium-free filter unit to remove any remaining particulate matter and potential bacterial contaminants.

***Note:*** This step is only required when purifying therapeutic proteins for *in vivo* injection to ensure sterility and prevent bacterial contamination. Ensure all tools, including pipette tips and tubes used during sample preparation, are sterilized. Applications involving quantification or other *in vitro* assays, this step is not necessary.

13. NPN-mediated purification.
  a. Aliquot filtered protein extract into sterile tubes.
  b. Add NPN to the protein extract at the desired concentration (e.g., 1 unit of NPN can be used to purify target proteins from 2 to 5 mL of total soluble protein extract).
  c. Incubate the mixture at 4°C for 15 minutes on shaker.
  d. Filter out the unbound fraction.
  e. Wash three times with sterilized TBS buffer.
  f. Resuspend in an appropriate buffer for subsequent in vivo injection or in vitro assays.

***Note:*** During the incubation step, maintaining 4°C is critical, as it effectively suppresses the activity of endogenous plant proteases, thereby reducing degradation of the target protein. In addition, the low-temperature condition helps minimize nonspecific binding.

## Expected outcomes

**Figure 2:**
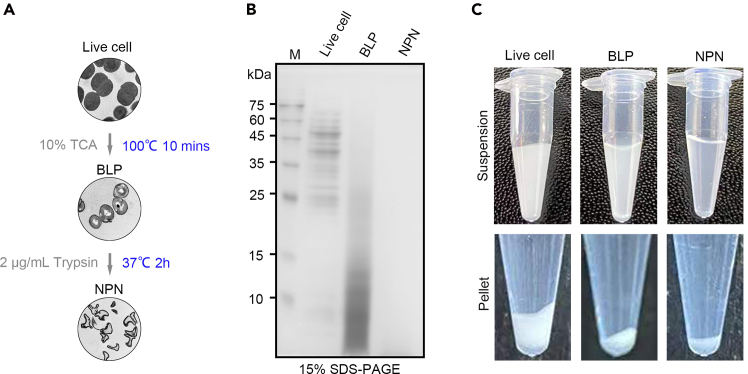
Structural and protein composition analysis of NPNs (A) Schematic illustration of NPN preparation and corresponding transmission electron microscopy images. (B) SDS-PAGE and Coomassie Brilliant Blue staining of live cells, BLPs, and NPNs. (C) Equal amounts of *L. sakei* cells (4 mL, OD_600_ = 1) were processed into live cells, BLPs, and NPNs, respectively, and the resuspension status of each preparation (100 μL) was visualized in PCR tubes.

**Figure 3:**
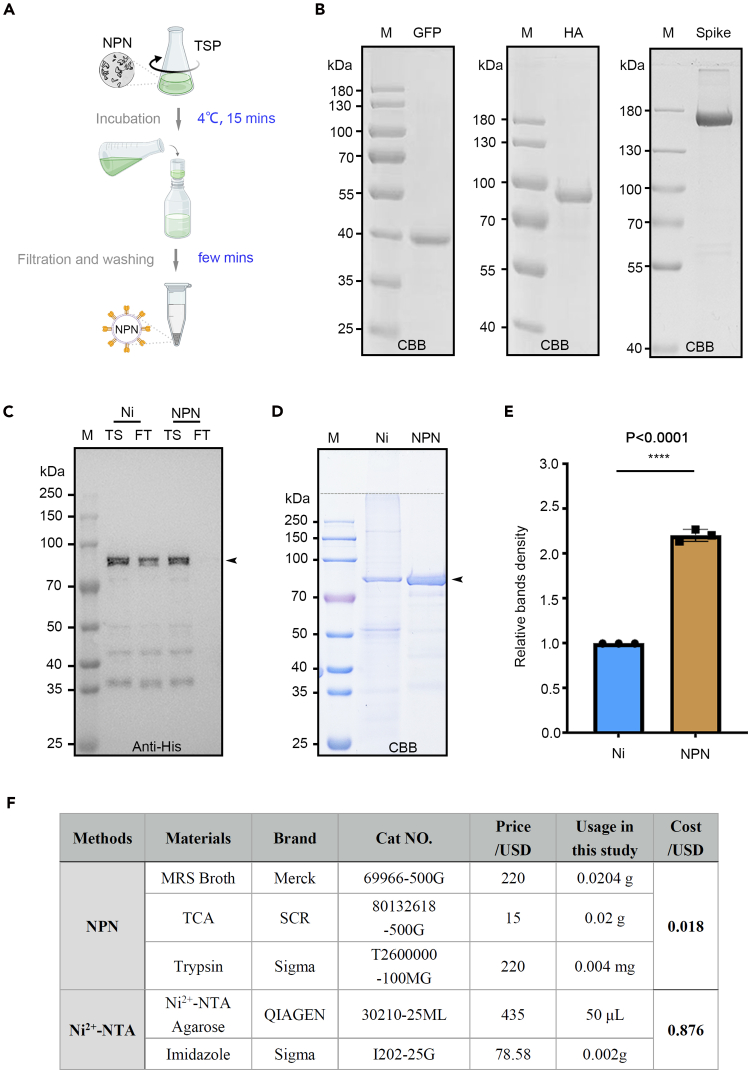
NPN-mediated proteins purification (A) Schematic illustration of NPN-mediated proteins purification. (B) SDS-PAGE and Coomassie Brilliant Blue staining of NPN conjugated target proteins. (C) SDS-PAGE and western blotting test for unbound proteins. M, protein marker; TS, total soluble proteins; FT, Flow through. Black arrow indicates the bands of HA-FD-SCpl7; Black arrow indicates the HA-FD-SCpl7 fractions. (D) SDS-PAGE and CBB staining test for bound proteins. M, protein marker; Ni, Ni^2+^-NTA beads after binding; NPN, NPN after binding. Black arrow indicates the bands of HA-FD-SCpl7; The quantification result of Ni^2+^-NTA and NPN-bound HA-FD-SCpl7 was shown in (E). The results are mean ± SD (n = 3). Statistical significance was analyzed using Student *t* test from the software, GraphPad Prism. (F) Cost comparation of NPN and Ni^2+^-NTA purification in this study.

The successful execution of this protocol is expected to yield uniform and clean natural peptidoglycan nanoparticles derived from LAB through sequential chemical and enzymatic treatments. Live cells, BLPs, and NPNs were examined by transmission electron microscopy (Figure 2A). The NPNs displayed an absence of residual subcellular structures. Further characterization by SDS-PAGE followed by Coomassie Brilliant Blue staining confirmed the absence of host protein contamination (Figure 2B). Notably, the concentration of NPNs was markedly lower compared with that of live cells or BLP (Figure 2C). Based on the NPN-mediated purification method for plant-derived recombinant proteins described above (Figure 3A), we constructed three recombinant proteins, GFP-FD-SCpl7, HA-FD-SCpl7, and Spike-FD-SCpl7 with a C-terminal 6×His tag. After NPN purification, SDS-PAGE and CBB staining revealed that all three target proteins exhibited high purity, with no detectable non-specific binding of endogenous plant proteins (Figure 3B).

To compare this approach with conventional nickel affinity chromatography, equal amounts of HA-FD-SCpl7 infiltrated plant tissue (0.1 g) were subjected to Ni^2+^-NTA purification (50 μL of resin) and NPN-mediated purification (2 units), respectively. The results showed that Ni^2+^-NTA failed to recover the full amount of target protein from total soluble protein, despite that 50 μL of resin theoretical binding capacity of 2.5 mg of His-tagged protein according to the instruction manual.^10^ Notably, NPNs could extract all target proteins from the total soluble fraction (Figure 3C). Furthermore, quantitative analysis demonstrated that the yield of purified protein obtained using NPNs was significantly higher and purer than that achieved by Ni^2+^-NTA purification (Figures 3D and 3E). Notably, the NPN purification process achieved an approximately 50-fold reduction in cost relative to the Ni^2+^-NTA method in this study (Figure 3F).

When applied to the purification of plant-derived recombinant antigens, the NPNs will demonstrate efficient and selective binding to target proteins engineered with affinity tags (e.g., SCpl7). This binding will enable rapid, chromatography-free protein purification directly from crude plant extracts in 15 minutes,^2^ significantly reducing downstream processing time and cost. Structural analysis by cryo-EM confirmed that the purified antigens preserved their trimeric architecture. Consistently, in vivo immunization studies revealed that NPN-conjugated GFP exhibited sustained release in mouse muscle for up to 10 hours, leading to a more than 500-fold increase in antibody titers.^2^ Moreover, the method will be validated using a diverse panel of antigens, including the hemagglutinin of influenza viruses, the spike protein of coronaviruses, the glycoprotein B of herpesviruses, and the G protein of rhabdoviruses previously tested in our laboratory, thereby demonstrating its robustness and broad applicability. Overall, this protocol will offer a practical and efficient platform for the production and purification of plant-based subunit vaccines and therapeutic proteins.

## Limitations

This purification system relies on the use of a trimeric affinity tag (e.g., FD) to enable strong and selective binding between plant-derived antigens and natural peptidoglycan nanoparticles; thus, it may not be applicable to untagged or monomeric proteins without prior engineering. Additionally, the binding efficiency can vary depending on the size and surface accessibility of the recombinant proteins, potentially requiring individual optimization for different targets. Finally, although antigen structure and immunogenicity appear to be retained, further in vivo studies are needed to fully evaluate the safety, stability, and adjuvant performance of the NPN-antigen complexes across different administration routes and animal models.

## Troubleshooting

### Problem 1

The preparation of NPNs was unsuccessful.

### Potential solution

The preparation of NPNs involves two main steps: TCA heat treatment and trypsin digestion. The initial TCA treatment must be thorough to remove the S-layer and surface proteins of Lactobacillus cells, thereby creating optimal conditions for subsequent trypsin cleavage.

### Problem 2

NPN binding efficiency is low.

### Potential solution

In our current assays, the binding efficiency of the three tested proteins to NPNs (1 unit) ranged from approximately 2–7 μg. Values below this range may indicate improper folding or low expression of the target protein, which negatively affects binding. In such cases, redesign and optimization of the target protein are recommended, such as incorporating solubility-enhancing tags or introducing appropriately sized linkers between different domains.

### Problem 3

Non-specific binding to NPN.

### Potential solution

The appearance of nonspecific bands may arise from two possible causes: (i) fragmentation of the target protein band, or (ii) nonspecific binding of host proteins. To address the first scenario, an adequate amount of protease inhibitors should be added during total soluble protein extraction and incubation. For the second, it is important to maintain incubation at low temperature and limit the duration to within 15 minutes.

## Resource availability

### Lead contact

Further information and requests for resources and reagents should be directed to and will be fulfilled by the lead contact, Shi-Jian Song (songshijian@caas.cn).

### Technical contact

Technical questions on executing this protocol should be directed to and will be answered by the technical contact, Hai-Ping Diao (haipingdiao@163.com).

### Materials availability

This study did not generate new unique reagents.

### Data and code availability

This study did not generate/analyze datasets and codes.

## Acknowledgments

This research was supported by Science and Technology Foundation of Beijing Life Science Academy (2024200CB0110) and the Natural Science Foundation of Shandong Province (ZR2024QC068).

## Author contributions

S-.J.S. contributed to the conception of the study and revised the manuscript. H.-P.D. and H.-X.M. contributed significantly to the analysis and experiments with the help of X.-J.X. S.-Y.R. wrote the draft with the help of S.-J.S. and Y.-F.G.

## Declaration of interests

The authors declare no competing interests.
